# The role of different exercises in irisin, heat shock protein 70 and some biochemical parameters

**DOI:** 10.5937/jomb0-31551

**Published:** 2022-04-08

**Authors:** Taner Akbulut, Vedat Cinar, Suleyman Aydin, Meltem Yardim

**Affiliations:** 1 Firat University, Faculty of Sport Sciences, Elazig, Turkey; 2 Firat University, Faculty of Medicine, Department of Medical Biochemistry, Elazig, Turkey; 3 Yerkoy State Hospital, Faculty of Sport Sciences, Department of Medical Biochemistry, Yozgat, Turkey

**Keywords:** biochemistry, exercise, irisin, lipid profile, muscle damage, biohemija, vežbe, irisin, lipidni profil, oštećenje mišića

## Abstract

**Background:**

The aim of the study was to determine the effects of different and regularly applied exercise programs on irisin, heat shock protein 70 and some biochemical parameters.

**Methods:**

120 male university students participated in the study. Participants were divided into 4 equal groups as control (C), resistance exercise group (RE), high intensity interval (HIIT) and aerobic exercise group (AE). While the control group did not perform any exercise, the pre-determined exercise programs were applied to the other groups for 8 weeks and 3 days in a week. Blood samples were taken from all participants before and after the exercise program. Cholesterol, High-density Lipoprotein (HDL) and Low-density Lipoprotein (LDL) cholesterol, triglyceride (TG), Creatine kinase (CK), Lactate dehydrogenase (LDH), Irisin and Heat shock protein 70 (HSP70) levels were analyzed in blood samples.

**Results:**

It is determined that there are significant differences in pre-posttest values of the AE group's LDH, cholesterol, HDL-cholesterol, TG and HSP 70 levels, HIIT group's CK, LDH, Cholesterol, HDL-cholesterol, TG, Irisin and HSP70 levels and RE group's CK, LDH, Cholesterol, LDL-cholesterol, TG and Irisin levels (p<0.05).

**Conclusions:**

It can be said that exercise can provide improvements in lipid profile, changes in HSP70 levels may vary depending on muscle damage, the increase of irisin due to exercise.

## Abstract (sr)

**Uvod:**

Cilj ove studije je bio sa se utvrde efekti različitih i redovno primenjivanih programa vežbanja na irisin, protein toplotnog šoka 70 i neke biohemijske parametre.

**Metode:**

U studiji je učestvovalo 120 univerzitetskih studenata. Učesnici su podeljeni u 4 jednake grupe kao kontrola (K), grupa vežbi otpora (RE), interval visokog intenziteta (HIIT) i grupa aerobnih vežbi (AE). Dok kontrolna grupa nije izvela nijednu vežbu, unapred određeni programi vežbanja primenjeni su na ostale grupe 8 nedelja i 3 dana u nedelji. Uzorci krvi uzeti su od svih učesnika pre i posle programa vežbanja. U uzorcima krvi je analiziran nivo holesterola, lipoproteina visoke gustine (HDL) i lipoproteina niske gustine (LDL), triglicerida (TG), kreatin kinaze (CK), laktat dehidrogenaze (LDH), irisina i proteina toplotnog šoka 70 (HSP70).

**Rezultati:**

Utvrđeno je da postoje značajne razlike u vrednostima pre-posttesta nivoa LDH, holesterola, HDL-holesterola, HDL-holesterola, TG i HSP 70 u AE grupi, CK, LDH, holesterola, HDL-holesterola, TG, irisina i HSP70 u grupi HIIT nivoi CK, LDH, holesterola, LDL-holesterola, TG i irisina u grupi RE (p <0,05).

**Zaključak:**

Može se reći da se vežbanjem može poboljšati profil lipida, promene nivoa HSP70 mogu varirati u zavisnosti od oštećenja mišića i povećanja irisina usled vežbanja.

## Introduction

Exercise is defined as planned, structured, repetitive, continuous activities that aiming to improve one or more elements of physical fitness [1]. This development occurs mostly by the activation of skeletal muscles in the organism. Depending on the characteristics and content of the training, the exercises can be performed in various types as aerobic or anaerobic [2]. In general, it is known that exercise has many effects on the human organism. Exercise causes an increase or decrease in the levels of some hormones. These changes occur with the activation of the glands [3]. The recently discovered protein that causes white adipose tissue to turn into brown adipose tissue and enables it to be effective in energy metabolism is called "irisin". Boström et al. [4] have reported that there is an increase in irisin values due to the overexpression of Fibronectin Type III Domain Containing 5 (FNDC5) in the liver of overweight (obese) rats. It has been stated that this increase causes browning of white adipose tissue and decreases in body weight. They also revealed that better oxygen consumption leads to glucose tolerance and insulin sensitivity. It is known that irisin is released into the circulation from many different parts and these are mainly skeletal and cardiac muscle, adipose tissue, brain, kidney, lung, liver and stomach [5]. However, it is stated that the synthesis of the irisin mainly takes place in the skeletal and cardiac muscles and adipose tissue [6]. In a study conducted on this field, it has been shown that resistance training applied 3 days a week for 12 weeks improves muscle functions and increases the level of irisin [7]. There are Heat shock proteins that have different roles in living organisms. It is known that these proteins are located in different sections of the cell. Since cells have common structural and functional properties, they contain members of different HSP families with distinct stress excitability. One of them is HSP70. HSP70 is known to be generally highly induced by stress such as oxidative stress and elevated temparature [8]. In an important study conducted in this topic, Cumming et al. [9] revealed that increases in HSP 70 level occur 48 hours after resistance exercises. It is stated that exercise has biochemical effects besides these effects, and it can have significant effects on muscle damage [10] [11] and blood lipid profiles depending on the type and intensity of the exercise [12]. It is known that physical activity and exercises have many positive effects on the human organism. Regular exercise helps weight control, supports body composition and reduces cardiovascular risks. The number of studies supporting these positive effects is increasing day by day. While the level of the irisin increases with the exercise factor, it is important how heat shock proteins will respond to this. The aim of this study is to examine the changes of irisin, HSP 70 and some biochemical parameters (blood lipid profile and muscle damage markers) of different exercises for eight weeks.

## Materials and Methods

### Participants

120 healthy students between the ages of 19-24 who study at Firat University, do not exercise regularly, stay in the same dormitory and are subject to the same nutrition program participated in the study. The research was conducted on a voluntary basis. Therefore, individuals who wanted to participate voluntarily and did not have any disability for exercise practices were included in the study. The research was explained to the participants as detail and approval was obtained from Firat University Non-Invasive Research Ethics Committee before starting the study (09/20-08.06.2017).

### Research Groups and Exercise Programs

#### Control Group (n: 30)

The group that did not have any exercise program.

#### Aerobic Exercise Group (n: 30)

An exercise program with 60-75% intensity (maximal heart rate) of the Karvonen method was applied to the participants for eight weeks, 3 days a week with continuous running method. Each exercise unit consisted of 10 minutes of warm-up, 40 minutes of main phase and 10 minutes of cool-down exercises. The training intensity of the group was designed according to the karvonen method by checking the maximal heart rate every week regularly.

#### High Intensity Interval Training Group (n: 30)

Participants were given an exercise program with the HIIT (High-intensity interval training) method 3 days a week for eight weeks. According to the principle of Tabata Type High Intensity Interval Training, each exercise unit was performed in 8 repetitions, with 15 minutes of warm-up, 20 seconds of ultraintensive loading, followed by 10 seconds cool-down [13].

#### Resistance Exercise Group (n: 30)

The trainings were carried out for eight weeks with 65-75% of the 1RM (Maximum repetition) values of the participants. Exercises were performed 3 days a week in a circular training format and exercise program consisting of 10 exercises covering both upper and lower extremity muscle groups. Each exercise unit was applied for 15 minutes of warm-up, each movement 3 sets and 8-10 repetitions. The weights used were arranged by checking each week according to the 1 maximum repetitions of the participants.

### Analysis of Biochemical Parameters

Blood samples taken for blood lipids (Chole sterol, HDL-cholesterol, LDL-cholesterol, Triglyceride), CK (Creatine Kinase), LDH (Lactate dehydrogenase), values of the participants in the study were centrifuged at 4000 rpm for 5 minutes. Blood samples were stored at -80 °C until the day of analysis. Serum samples were analyzed with Olympus autoanalyzer (AU 2700; Hamburg, Germany).

### Analysis of Irisin and HSP 70

Irisin (Catalog no: 201-12-5328 Sunred, Biological Technology Co., Ltd., Shanghai, CHINA) and HSP70 levels (Catalog no: 201-12-1814, Sunred, Biological Technology Co., Ltd., Shanghai, CHINA) were analyzed by ELISA method in accordance with the working procedures specified in the kit catalogs. Irisin measuring range of ELISA kit: 0.2-60 ng/mL sensitivity was 0.157 ng/mL, HSP 70; 0.5-150 ng/mL sensitivity was 0.458 ng/mL. Irisin and HSP 70 Intra-Assay: CV <10%, Inter-Assay: CV was <12%. Automatic washer Bio-Tek ELX50 (BioTek Instruments, USA) was used for plate washing, ChroMate for absorbance readings, Microplate Reader P4300 devices (Awareness Technology Instruments, USA) and test results were reported as ng/mL.

### Statistical analysis

SPSS 22.0 package program was used to analyze the data obtained from the research. Data distribution was checked Kolmogorov Smirnov test. The distribution was normal. Thus Paired Samples t test was performed to determine the intra-group differences that occurred before and after the exercise protocol and One Way ANOVA analysis was performed to reveal the differences between groups. Tukey test, one of the Post Hoc tests, was applied to determine which group caused the difference. Statistical significance was accepted as p <0.05 for all tests. The data were presented in Tables.

## Results

In Table 1, it was determined that there was significant differences between the pre-test post-test values of LDL-cholesterol in the control group. There were significant differences cholesterol, HDL-cholesterol, triglyceride levels of aerobic exercise group and HIIT group. In addition significant differences were found in cholesterol, LDL-cholesterol, triglyceride levels of the resistance exercise group (p<0.05). When the percentage change of these differences is examined; it was observed that the greatest change in cholesterol and triglyceride levels occurred in the aerobic exercise group, HDL-cholesterol in the HIIT group, and LDL-cholesterol in the resistance exercise group.

**Table 1:** Blood Lipids Levels of the Research Groups. ^**^There is a significant difference (P<0.01). The difference between groups with different letters in the same column is significant (a,b,c,d)

Parameters	Groups	Pretest	Posttest	Changes (%)	Intra-group	Inter-groups
		Mean±Sd	Mean±Sd		p	p
Cholesterol (mmol/L)	Control	4.2030±0.80	4.2056±0.80	0.06	0.620	0.089
	Aerobic	4.5255±0.71	4.1867±1.03	-7.48	0.020^*^	
	HIIT	4.1996±0.80	3.9927±0.67	-4.92	0.010^**^	
	Resistance	3.9979±0.70	3.7411±0.59	-6.42	0.022^*^	
HDL-Cholesterol <br>(mmol/L)	Control	1.0868±0.20	1.0843±0.20	-0.23	0.083	0.219
	Aerobic	1.0912±0.20	1.1732±0.21	7.50	0.001^**^	
	HIIT	0.9852±0.15	1.1637±0.17	18.11	0.000^**^	
	Resistance	1.0879±0.14	1.1150±0.15	2.51	0.310	
LDL-Cholesterol <br>(mmol/L)	Control^b^	2.4238±0.86	2.4326±0.86	0.36	0.002^**^	0.009^**^
	Aerobic^b^	2.5757±0.54	2.5808±0.88	0.20	0.965	
	HIIT^b^	2.4334±0.86	2.2886±0.57	-5.60	0..236	
	Resistance^a^	2.2014±0.61	1.9309±0.66	-12.28	0.004^**^	
Triglyceride (mmol/L)	Control^b^	1.7518±0.75	1.7310±0.77	-1.18	0.084	0.004^**^
	Aerobic^a^	1.7496±0.77	1.2174±0.40	-30.41	0.000^**^	
	HIIT^a^	1.8301±0.85	1.4067±0.44	-23.13	0.021^*^	
	Resistance^a^	1.6249±0.48	1.4368±0.43	-11.57	0.041^*^	

When Table 2 is evaluated; It was determined that there were significant differences between pre-test post-test values of CK in control group, LDH level in aerobic exercise group and both CK and LDH levels in HIIT group and resistance exercise group (p <0.05). The biggest change was seen in the Resistance exercise group in both parameters as a percentage. A statistically significant difference was found between the posttest CK and LDH levels between the groups (p <0.05). While this difference is due to the resistance exercise group at the CK level (p: 0.01), it is due to the resistance exercise group and interval group at the LDH level (p: 0.00). There was also a difference in LDH level between resistance exercise and HIIT group (p: 0.00).

**Table 2:** Muscle Damage Markers of the Research Groups. ^**^There is a significant difference (P<0.01), The difference between groups with different letters in the same column is significant (a,b,c,d)

Parameters	Groups	Pretest	Posttest	Changes (%)	Intra-group	Inter-groups
		Mean±Sd	Mean±Sd		p	p
CK (U/L)	Control^b^	235.10±136.93	237.23±135.85	0.90	0.000^**^	0.004^**^
	Aerobic^b^	234.70±137.80	264.90±165.01	12.86	0.195	
	HIIT^b^	237.33±90.08	387.30±110.06	63.19	0.000^**^	
	Resistance^a^	231.17±121.6	417.87±185.59	80.76	0.007^**^	
LDH (U/L)	Control^d^	173.57±10.23	176.17±8.89	1.49	0.058	0.000^**^
	Aerobic^d^	170.87±8.39	178.30±16.66	4.34	0.011^*^	
	HIIT^c^	176.00±12.76	202.23±17.41	14.90	0.000^**^	
	Resistance^ab^	171.50±12.47	239.47±30.17	39.63	0.000^**^	

When Table 3 is examined, it is determined that there was a difference between the pre-test post-test values of Irisin in all exercise groups (p <0.05), and in the HSP70 level, there was a significant difference in all groups except the resistance exercise group (p <0.05). When the differences was calculated as percentage, it was determined that the greatest change in irisin level occurred in the HIIT group and at the HSP70 level in the aerobic exercise group. In addition, it was found that there was a significant difference between the groups in both irisin (p: 0.00) and HSP 70 posttest levels (p: 0.00).

**Table 3:** Irisin and HSP70 Levels of the Research Groups. ^**^There is a significant difference (P<0.01), The difference between groups with different letters in the same column is significant (a,b,c,d)

Parameters	Groups	Pretest	Posttest	Changes (%)	Intra-group	Inter-groups
		Mean±Sd	Mean±Sd		p	p
Irisin (ng/mL)	Control^a^	13.91±3.05	14.39±4.90	3.45	0.464	0.000^**^
	Aerobic^a^	8.36±2.41	11.41±4.42	36.48	0.000^**^	
	HIIT^b^	17.32±4.54	26.78±3.49	54.61	0.000^**^	
	Resistance^b^	19.82±6.57	26.02±7.65	31.28	0.000^**^	
HSP70 (ng/mL)	Control^a^	25.70±4.25	24.83±4,41	-3.38	0.000^**^	0.000^**^
	Aerobic^b^	39.44±8.74	7.66±3.26	-80.57	0.000^**^	
	HIIT^b^	20.03±6.98	5.54±1.93	-72.34	0.000^**^	
	Resistance^c^	16.40±5.25	15.09±2.19	-7.98	0.156	

## Discussion

In the present study, it was determined that exercises performed in different types (resistance, high intensity interval and aerobic) during eight weeks caused significant changes in blood lipid profile, enzymes associated with muscle damage, irisin and HSP 70 levels. When the changes in the lipid profile are examined; It was determined that HDL-cholesterol levels increased in all exercise groups. As with the change in cholesterol levels, the change in HDL-cholesterol is thought to be due to the positive effects of exercise. When LDL-cholesterol values were examined, it was observed that there was a significant decrease in the resistance exercise group. In addition triglyceride levels changed significantly in all exercise groups. It can be said that this situation is due to the positive effects that exercise can create on triglycerides. When the effects of exercise on lipid profile are investigated, it is seen that there are many studies. 54 healthy men voluntarily participated in one of these studies, it was stated that aerobic and anaerobic training programs applied 3 days a week for 8 weeks caused an increase in HDL-cholesterol, however it led to a decrease in LDL-cholesterol and consequently had positive effects on lipid profile [14]. In another study, Wang and Xu [15] concluded that aerobic exercise led to an improvement in lipid profile in a review study aimed at evaluating the effect of aerobic exercise on lipids and lipoproteins. Similarly, Yao et al. [16] examined the effects of aerobic and resistance exercises on liver enzymes and blood lipids in non-alcoholic fatty liver patients. As a result of the research, they stated that similar to current study results, there was a change in HDL-cholesterol level in the resistance exercise group, and there were changes on both HDL-cholesterol and triglyceride levels in the aerobic exercise group. The other study, the effects of traditional resistance training and high-intensity resistance training were investigated on lipid profile in the elderly population. As a result of the research, it has been determined that both resistance exercise groups have positive effects on blood lipids just as in the present study [17]. When the effects of exercise on markers of muscle damage are evaluated; it was observed that CK values increased significantly in the resistance exercise group and high-intensity interval group. When the differences between the groups were determined, it was determined that the resistance exercise group reached the highest value. It is thought that this situation is caused by the intensity of the exercise and the use of extra weights in the resistance exercise group. There were also increases in LDH values in all exercise groups. The greatest increase was determined in the resistance exercise group, just as in CK. This result suggests that exercise may have negative effects in the transition to regular exercise in individuals who have not exercised regularly before. As a result, it is seen that the effects of different exercise types on muscle damage markers differ according to their intensity. When studies on muscle damage are analyzed; as in the resistance exercise group in current study, it is also seen as a result of a study that was applied acutely that strength training applied maximally caused muscle damage significantly [18]. In another study, Penailillo et al. [19] have reported that both concentric and eccentric cycling exercises caused increases at a low level in CK levels. In a study conducted by Brentano et al. [20] it was stated that strength training with 5 sets and 8-10 repetitions prepared according to the super set method significantly increased the CK level. Similarly, in a study involving 16 volunteer participants who did not do sports, a resistance exercise program consisting of 3 sets of 10 repetitions and 5 exercises was applied to the participants. As a result of this research applied acutely, it has been determined that resistance exercises cause muscle damage and cause significant increases in CK and LDH values [21]. Pal et al. [22] have examined oxidative stress and muscle damage that occur in response to high intensity exercise in young people. CK and LDH levels were analyzed in the blood samples taken 24 and 48 hours after the applied high intensity treadmill run. As a result, they found that an increase in enzyme levels associated with muscle damage after high intensity exercise. Similar results were obtained in present study, although it was applied for a long time. Current study shows that it was determined that irisin level increased in all exercise groups. In comparisons between groups, it was determined that the lowest increase was in the control group. From this point of view, it is thought that the effect of exercise on the irisin level is closely related to the type, intensity, frequency, scope of the exercise, and the age and training level of the participants. It can be said that these factors can seriously affect the results of the studies on irisin. Previous studies are explain the relationship and interaction between exercise and irisin. In one of these studies, it was stated that regular exercise led to the activity of the irisin, although the effects of exercise and physical activity at the plasma irisin level were contradictory [23]. In addition, it is stated that the level of irisin is higher in young people than in the elderly, and higher in physically active individuals than in sedentary individuals [24]. Kim et al. [7] examined the effects of exercise on the irisin level on both rats and humans in their study. For this purpose, resistance (climbing exercise) applied to 19-week old rats 3 days a week for 12 weeks, and resistance (elastic band) exercises were applied to people over 65 years old 2 days a week for 12 weeks. As a result of the research, both animals and humans revealed that there was an increase in strength and an irisin levels. In another study, Reisi et al. [25] do resistance exercises change the level of the irisin? Based on the question, they have investigated that the effect of 3 sets with 5 repetitive resistance exercises applied for 8 weeks, 3 days a week on the level of irisin in rats. As a result of the study, they concluded that eight-week resistance exercises increased the level of irisin. The present study shows that HSP70 level did not change in the Resistance exercise group, but decreased in all other groups. This result shows that especially the aerobic and high-intensity interval groups adapt to the exercises more quickly. Depending on this adaptation, decreases in HSP70 levels are observed. In addition, it is thought that the use of additional weights in the resistance exercise group causes a stress in the human organism and causes this decrease to be less. As a result of the literature review, it is seen that the number of studies investigating the effect of exercise on HSP70 level is limited. Previous studies show that different results are obtained regarding the relationship between regular exercise and HSP70. Although there are still conflicting results, the general opinion is that regular exercise reduces or does not change the level of HSP70. This situation reveals the importance of the type, duration, frequency, intensity and scope of the exercise. The research group is also important. Considering the studies on HSP 70, it was concluded that 11-week strength training did not cause an increase in the trapezius muscle while there was a significant increase on HSP70 level at the vastus lateralis muscle in the biopsy samples [26]. Considering the muscle groups with and without an increase, the part of body included in the resistance exercise program, the number of repetitions and the exercise intensity could gain importance. In addition, the resting period is another important issue [27]. In another study, Krause et al. [28] investigated the HSP70 level in healthy elderly individuals with combined resistance exercises using body weight and elastic bands. Within the scope of the research, participants were divided into four groups: placebo, protein supplement, placebo + exercise, and protein supplement + exercise. As a result of exercises that were continued for 12 weeks and 3 days a week, it was stated that there was no statistically significant difference in the group that exercised and was given placebo. This result is similar to the resistance exercise group of the present study.

## Conclusion

As a result, considering all exercise groups; in parallel with the literature, it can be stated that regular exercise has positive positive effects in human organism. Within the scope of the current study, it was said that regular exercise programs produced positive results in blood lipids, which are considered as cardiovascular risk markers. In addition, it has been observed that irisin levels increase with exercise. It could be said that HSP 70 levels decrease especially with aerobic exercise and high-intensity interval exercise, and that stress decreases in response to exercise and hence the release of HSP 70 level decreases.

*Support*: There are no financial support. *Author’s note*: This article was obtained from doctoral thesis of Taner Akbulut.

## Dodatak

### Conflict of interest statement

All the authors declare that they have no conflict of interest in this work.

## References

[1] Ozer M K (2006). Physical Fitness.

[2] Zorba E, Saygın O (2009). Assessing Physical Activity Patterns and Physical Fitness.

[3] Fox E L, Bowers R W, Foss M L (2012). Essantials of Physical Education and Sport.

[4] Boström P, Wu J, Jedrychowski M P, Korde A, Ye L, Lo J C, et al. (2012). A PGC1-a-Dependent Myokine That Drives Brown-Fat-Like Development of White Fat and Thermogenesis. Nature.

[5] Ilhan M (2018). Effects of Physical Activity and Nutrition on Fibroblast Growth Factor 21 and Irisin in Non-Alcoholic Fatty Liver Disease (Master Thesis).

[6] Aydin S (2014). Three New Players in Energy Regulation: Preptin, Adropin and Irisin. Peptides.

[7] Kim H J, So B, Choi M, Kang D, Song W (2015). Resistance Exercise Training Increases the Expression of Irisin Concomitant with Improvement of Muscle Function in Aging Mice and Humans. Experimental Gerontology.

[8] Rylander M N, Feng Y, Bass J, Diller K R (2005). Thermally Induced Injury and Heat-Shock Protein Expression in Cells and Tissues. Ann N Y Acad Sci.

[9] Cumming K T, Paulsen G, Wernbom M, Ugelstad I, Raastad T (2014). Acute Response and Subcellular Movement of HSP27,aB-Crystallin and HSP70 in Human Skeletal Muscle After Blood-Flow-Restricted Low-Load Resistance Exercise. Acta Physiologica (Oxf).

[10] Coban O (2011). Investigation of Effects of Regular Coenzym Q10 Supplementations on Muscle Damage and Trace Elements of Wrestlers (PhD Thesis).

[11] Byrne C, Eston R (2002). The Effect of Exercise-Induced Muscle Damage on Isometric and Dynamic Knee Extensor Strength and Vertical Jump Performance. J Sports Sci.

[12] Cinar V, Akbulut T, Kilic Y, Ozdal M, Sarikaya M (2018). The Effect of 6-Week Zinc Supplement and Weight Training on the Blood Lipids of the Sedentaries and Athletes. Cell Mol Biol (Noisy-le-grand).

[13] Tabata I, Nishimura K, Kouzaki M, Hirai Y, Ogita F, Miyachi M, et al. (1996). Effects of Moderate-Intensity Endurance and High-Intensity Intermittent Training on Anaerobic Capacity and VO2MAX. Med Sci Sports Exerc.

[14] Koc H, Tamer K (2008). The Effects of Aerobic and Anaerobic Trainings on Lipoprotein Levels. Journal of Health
Sciences.

[15] Wang Y, Xu D (2017). Effects of Aerobic Exercise on Lipids and Lipoproteins. Lipids in Health and Disease.

[16] Yao J, Meng M, Yang S, Li F, Anderson R M, Liu C, et al. (2018). Effect of Aerobic and Resistance Exercise on Liver Enzyme and Blood Lipids in Chinese Patients with Nonalcoholic Fatty Liver Disease: A Randomized Controlled Trial. Int J Clin Exp Med.

[17] Juhas I, Skof B, Popović D, Matić M, Janković N (2020). Effects of an eight-week exercise program on parameters of the lipid profile of female students. J Med Biochem.

[18] Hazar S, Erol E, Gokdemir K (2006). Relationship Between the Muscle Damage and Delay Onset Muscle Soreness After Strength Training. Gazi BESBD.

[19] Peñailillo L, Blazevich A, Numazawa H, Nosaka K (2013). Metabolic and Muscle Damage Profiles of Concentric versus Repeated Eccentric Cycling. Medicine and Science in
Sports and Exercise.

[20] Brentano M A, Umpierre D, Santos L P, Lopes A L, Radaelli R, Pinto R S, et al. (2017). Muscle Damage and Muscle Activity Induced by Strength Training Super-Sets in Physically Active Men. J Strength and Condition Res.

[21] Barquilha G, Silvestre J C, Motoyama Y L, Azevedo P H S M D (2018). Single Resistance Training Session Leads to Muscle Damage Without Isometric Strength Decrease. J Human Sport and Exer.

[22] Pal S, Chaki B, Chattopadhyay S, Bandyopadhyay A (2018). High-Intensity Exercise Induced Oxidative Stress and Skeletal Muscle Damage in Postpubertal Boys and Girls: A Comparative Study. J Strength and Condition Res.

[23] Palacios-González B, Vadillo-Ortega F, Polo-Oteyza E, Sánchez T, Ancira-Moreno M, Romero-Hidalgo S, et al. (2015). Irisin Levels Before and After Physical Activity Among School-age Children with Different BMI: A Direct Relation with Leptin. Obesity (Silver Spring).

[24] Huh J Y, Mougios V, Kabasakalis A, Fatouros I, Siopi A, Douroudos I I, et al. (2014). Exercise-Induced Irisin Secretion Is Independent of Age or Fitness Level and Increased Irisin May Directly Modulate Muscle Metabolism Through AMPK Activation. J Clin Endocrinol Metab.

[25] Reisi J, Ghaedi K, Rajabi H, Marandi S M (2016). Can Resistance Exercise Alter Irisin Levels and Expression Profiles of FNDC5 and UCP1 in Rats?. Asian J Sports Med.

[26] Paulsen G, Hanssen K E, Rønnestad B R, Kvamme N H, Ugelstad I, Kadi F, et al. (2012). Strength Training Elevates HSP27, HSP70 and aB-Crystallin Levels in Musculi Vastus Lateralis and Trapezius. Eur J Appl Physiol.

[27] Judge L W, Burke J R (2010). The Effect of Recovery Time on Strength Performance Following a High-Intensity Bench Press Workout in Males and Females. Int J Sports Physiol Perform.

[28] Krause M, Crognale D, Cogan K, Contarelli S, Egan B, Newsholme P, et al. (2019). The Effects of a Combined Bodyweight-Based and Elastic Bands Resistance Training, with or Without Protein Supplementation, on Muscle Mass, Signaling and Heat Shock Response in Healthy Older People. Exp Gerontol.

